# Meta-Learning for Few-Shot Plant Disease Detection

**DOI:** 10.3390/foods10102441

**Published:** 2021-10-14

**Authors:** Liangzhe Chen, Xiaohui Cui, Wei Li

**Affiliations:** 1 School of Artificial Intelligence and Computer Science & Jiangsu Key Laboratory of Media Design and Software Technology & Science Center for Future Foods, Jiangnan University, Wuxi 214122, China; lzchen@stu.jiangnan.edu.cn; 2 School of Cyber Science and Engineering, Wuhan University, Wuhan 430072, China; xcui@whu.edu.cn

**Keywords:** food security, plant disease detection, convolutional neural networks, few-shot, meta-learning

## Abstract

Plant diseases can harm crop growth, and the crop production has a deep impact on food. Although the existing works adopt Convolutional Neural Networks (CNNs) to detect plant diseases such as Apple Scab and Squash Powdery mildew, those methods have limitations as they rely on a large amount of manually labeled data. Collecting enough labeled data is not often the case in practice because: plant pathogens are variable and farm environments make collecting data difficulty. Methods based on deep learning suffer from low accuracy and confidence when facing few-shot samples. In this paper, we propose local feature matching conditional neural adaptive processes (LFM-CNAPS) based on meta-learning that aims at detecting plant diseases of unseen categories with only a few annotated examples, and visualize input regions that are ‘important’ for predictions. To train our network, we contribute Miniplantdisease-Dataset that contains 26 plant species and 60 plant diseases. Comprehensive experiments demonstrate that our proposed LFM-CNAPS method outperforms the existing methods.

## 1. Introduction

Food shortages may increase in many regions of the world. Coupled with pests and crop failures, food prices have soared. A lot of people may face severe hunger and death. In order to solve the food shortage, it is necessary to ensure the food security and sustainability. Due to pests, diseases [1,2], and lack of horticultural expertise [3,4,5], food yield loss is greater than 50% [6]. Food security is increasingly affected by crop production [7]. With the increase of agricultural intensification and the continuous strengthening of the agricultural industry chain, the risks related to viruses and pollution will increase. For the goal of global food security and sustainable development, by 2050, the current demand of crop disease detection needs to increase by 50% [8].

The traditional method of plant disease detection is manual inspection by farmers or experts. The method of plant disease diagnosis through optical observation of the symptoms on plant leaves incorporates a significantly high degree of complexity [2]. The method laboratory-based such as polymerase chain reaction (PCR), immunofluorescence (IF), and fluorescence in-situhybridization (FISH) require professional laboratory equipment and mass sampling work [9]. Due to this complexity and to the large number of cultivated plants and their existing phytopathological problems, manual plant disease detection can be time-consuming and expensive [10]. By contrast, images under analysis were obtained by employing cameras operating in the visible portion of the electromagnetic spectrum (400–700 nm). In this way, costly equipment or trained personnel are not required for obtaining the input data [11]. Therefore, future users of the developed protocol can acquire data through affordable/cost-effective, portable (thus in situ), and rapid means. With the development of computational systems in recent years, and in particular Graphical Processing Units (GPU) embedded processors, Convolutional Neural Networks (CNNs) [12] is often applied for image classification.

CNNs belong to a stackable feedforward neural network community [12]. The method of image classification through multi-layer CNNs is also called deep learning [13,14,15]. CNNs have good characterization learning ability, so they are mostly used for feature extraction, and the extracted features have the characteristics of translation invariance. The research on CNNs began in the 1980s and 1990s, and the time delay network and LeNet-5 were the earliest CNNs [12]. For a convolution operation, the essence is a traversal of the convolution kernel on the feature image. The convolution kernel will multiply and add the value at the corresponding position of the input feature image. In recent years, CNNs have been increasingly incorporated in plant phenotyping concepts. They have been very successful in modeling complicated systems, owing to their ability of distinguishing patterns and extracting regularities from data. Examples further extend to the variety identification in seeds [16] and in intact plants by using leaves [17]. Some research [18] collected and published the datasets of plant diseases that provided data sources for other methods. There are also some works [10] using image segmentation technology to separate the foreground and the background that can further improve the classification accuracy, and also solve the problem of poor performance on the online test.

Although the above deep learning methods have good performance related to plant disease detection, they still have the following problems. The first is the common problem of deep learning; model training requires a large amount of manually labeled datasets. The above-mentioned methods are currently based on the support of a large amount of data. Each category requires more than 1000 pictures. Data collection and marking require manpower and time. There is not enough data to support network training for plant pathogen variables in time, space, and genotype [19]. The second problem is that many of the more than 700 known plant viruses cause devastating diseases and often have wide host ranges. Barley yellow dwarf viruses (BYDV), for example, are distributed worldwide and infect over 150 species of the Poaceae, including most of the staple cereals—wheat, barley, oats, rye, rice, and maize [19]. It is unrealistic to identify all plant diseases at once through one task. However, the emergence of new tasks requires retraining the network. The above methods all limit the total number of categories for specific classification of several plant diseases. Every time a new task is encountered, based on traditional deep learning methods, it is necessary to rearrange the data and train the network to adapt to the task. For different sample numbers and image sizes, professional knowledge is needed to fine-tune the hyperparameters in the network structure. The last problem is the poor interpretability of the method. Compared with manual detection, experts can provide the basis for plant disease detection such as Oval-shaped irregular brown spots appearing on the leaves of plants with rust, and the leaf color on the leaves gradually becoming lighter, and, using fluorescence imaging, temporal and spatial variations of chlorophyll fluorescence were analyzed for precise detection of leaf rust and powdery mildew infections in wheat leaves at 470 nm [9]. Although the deep-learning-based methods show their effectiveness, it cannot explain their decisions and actions to human users. Therefore, the methods should give visual explanations to illustrate that our approach focuses on diseases’ classification. This paper proposed a meta-learning [20] method to solve the challenge of plant disease detection.

Meta-learning is the science of systematically observing how different machine learning approaches perform on a wide range of learning tasks, and then learning from this experience, or meta-data, to learn new tasks much faster than otherwise possible [21]. There is a lot of research on meta-learning. Meta-learning is transfer learning in a broad sense [22], which chooses data from different sources to train the network so that the model has a good classification effect on all kinds of tasks. Few-shot learning [23], which is the problem of making predictions based on a limited number of samples, is an important application direction of meta-learning. The network is trained through other multi-source and sufficient datasets, so that it can deal with the task with few training samples. There is a lot of research on few-shot learning. For example, CNAPS [24] and Simple-CNAPS [25] use forward propagation instead of back propagation to solve the problem of overfitting, modular adaptation method [26], and Meta Fine-Tuning [27], which is also called Cross-Domain Few-Shot learning, can be trained to perform both tasks across domains. There is also some research on metric learning [28] such as MatchingNet [29] and ProtoNet [23] to solve the problem of insufficient samples and the poor performance of the classifier. Although there have been many works on few-shot learning, most of the works are more theoretically focused, and do not focus on specific applications. Based on the previous work, this paper applied meta-learning to plant diseases detection.

## 2. Materials and Methods

In this section, initially, the datasets chosen for training and testing are introduced. Afterwards, the meta-learning method proposed for plant detection called LFM-CNAPS is presented. Finally, visual explanations technology called TAM is introduced.

### 2.1. Datasets

The key to few-shot plant disease detection lies in the generalization ability of the pertinent model when presented with novel disease categories. Thus, high-diversity datasets are necessary for training the model that can detect unseen plant diseases. In this paper, Meta-Dataset [30] and Miniplantdisease-Dataset are chosen for model training.

A Meta-Dataset [30] is composed of 10 public datasets including ILSVRC-2012 (ImageNet) [31], Omniglot [32], FGVC-Aircraft (Air-craft) [33], CUB-200-2011 (Birds) [34], Describable Textures (DTD) [35], QuickDraw [36], FGVCx Fungi (Fungi) [37], VGG Flower (Flower) [38], Traffic Signs (Signs) [39], and MSCOCO [40]. Meta-Dataset is comprised of multiple existing datasets that contains more than 110,000 few-shot classification tasks. The tasks span a variety of visual concepts (natural and human-made) and vary in how fine-grained the class definition is [30]. Through Meta-Dataset training, the models’ ability to leverage diverse training sources will be improved.

Miniplantdisease-Dataset proposed in this paper is composed of Apple foliar disease Dataset [41] and PlantVillage-Dataset [18]. The Apple foliar disease Dataset contains 3651 high-quality and real photos of various apple foliar diseases. The PlantVillage-Dataset has released more than 50,000 specialized images through the online platform Plantvillage [18]. The PlantVillage-Dataset contains various diseases per plant categories, while the Apple foliar disease Dataset only contains healthy and unhealthy two labels per plant categories. In keeping with a results report, the PlantVillage is divided into two parts with a ratio of 8:2 to compose Miniplantdisease-Dataset and test model, respectively. We report results using 48 plant diseases for training called in-domain Miniplantdisease-Dataset, reserving other 12 plant diseases for out-of-domain performance evaluation.

### 2.2. LFM-CNAPS

**Figure 1:**
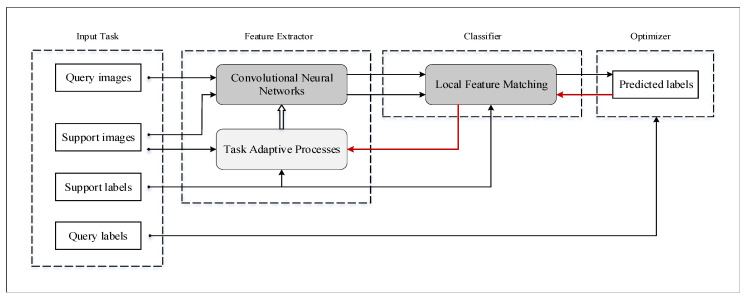
Flow chart of LFM-CNAPS. In the figure, the white solid squares are data variable, the black arrows are the data flow direction, the white hollow arrows are forward propagation, and the red arrows are backward propagation.

The method proposed in this paper to solve few-shot plant disease recognition is local feature matching conditional neural adaptive processes (LFM-CNAPS). As shown in Figure 1, it contains four main parts: input task, conditional adaptive feature extractor, and local feature matching classifier and parameters optimizer.

#### 2.2.1. Task

Miniplantdisease-Dataset contains many different meta-tasks. The model learns the generalization ability from meta-tasks. When facing new categories, the classification can be completed without changing the existing model. The Miniplantdisease-Dataset contains 60 plant disease categories, with multiple samples in each category. For any meta-task in Miniplantdisease-Dataset, five plant disease categories will be randomly selected, with five samples for each category (a total of 25 samples). These samples with their labels will be constructed as support images and support labels of meta-task. In addition, then extract 50 samples for test from the remaining five categories samples as query images and query labels. That is, the model is required to learn how to distinguish these five categories from 25 samples. Such a task is called a 5-way 5-shot problem.

#### 2.2.2. Conditional Adaptive Feature Extractor

The feature extractor chosen in this paper consists of two parts, a CNN framework named RESNET18 [42] and task adaptive processes [24]. Among them, RESNET18 is a stackable CNN layer with a batch normalization layer to prevent vanishing gradient and exploding gradient. The task adaptive process [24] is an effective method of impacting CNN intermediate variables to adapt the task [43]. The core of the task adaptive process is to choose forward propagation instead of back propagation to prevent overfitting due to few samples.

RESNET18 mainly contains CNNs and a Batch Normalization layer. The CNNs are essentially to do a dot product between the filter and the local area of the input data. The convolution kernel will multiply and add the value at the corresponding position of the input data, as shown in (1):

(1) $$Con\left(I_{x,y},K\right)=\sum_{c=1}^{C}\sum_{h=1}^{H}\sum_{w=1}^{W}I_{x+h,y+w,c}\times K_{h,w}$$

Among them, *I* represents the input data, *K* represents the convolution kernel, and *x* and *y* represent the position of the convolution kernel on the feature map *I*. *H*, *W*, and *C* respectively represent the length and width of the convolution kernel and the number of channels. The CNNs will extract specific local features according to the convolution kernel parameters. For CNNs, there are many hyperparameters such as the size of the convolution kernel, sliding step size, and the number of CNN layers. Different hyperparameter settings will have a great impact on the accuracy of the model. RESNET18 gives the hyperparameters suitable for most image feature extractions [42]. The core of RESNET18 [42] is stackable CNN layers, and the hyperparameters of the CNNs are fixed. On this basis, Batch Normalization layer is applied. In the Batch Normalization layer, such an operation is shown in (2):

(2) H(X)=F(X)+X

Among them, H(X) represents the Batch Normalization operation, F(X) represents the corresponding CNNs, and *X* represents the input. It can be seen intuitively that the process of Batch Normalization layer is to add the input *X* and the result of the CNNs. Batch Normalization effectively solves the problem of gradient vanishing and degradation caused by the network being too deep. Batch Normalization layers make deep network training possible.

The task adaptive process contains the task encoder and FILM layer [44]. The task encoder is composed of CNN layers and fully connected layers that take the support set as input and FILM layer parameters as output. The task encoder provides FILM layer parameters to make CNNs better adapt tasks. For traditional deep learning methods, back propagation is an important method of updating parameters. However, most deep learning methods require a large number of labeled samples. For a few-shot task, there are only a few labeled samples for training. Too few samples to update the parameters through back propagation will cause overfitting. That is, the accuracy of the training set is very high, and the result of the test set is very poor. To avoid this, the FILM layer [44] is proposed to perform affine transformation on the intermediate features of the CNNs, as shown in (3):

(3) $$\sum_{c=1}^{C}\sum_{x=1}^{X}\sum_{y=1}^{Y}I_{x,y,c}\times \gamma_{c}+\beta_{c}$$

Among them, *I* is the middle feature map, and *X*, *Y*, and *C* represent the length and width of the feature and the number of channels. γ and β are the parameters of the FILM layer and they are generated by the task encoder. The parameters updated by back propagation are proportional to the volume of the convolution kernel, and the forward propagation only needs to update the parameters that are proportional to the number of channels. Therefore, the depth of the overall network has not changed, but the number of updated parameters are reduced, avoiding the overfitting caused by few samples.

#### 2.2.3. Local Feature Matching Classifier

For image classification methods, classifiers are indispensable [45]. For the traditional method, after handcrafted features are extracted, a separate classifier such as SVM is needed. For deep learning, a fully connected layer and activation function are generally chosen as a classifier. For SVM, the parameters need to be trained separately [46]. For deep learning classifier that can be trained end-to-end, the fully connected layer contains hundreds of parameters that need to be optimized. When samples are not enough, parameter optimization can be difficult. Therefore, this paper chooses metric learning as the classifier for few-shot plant disease detection.

The obvious advantage of the metric learning classifier is that there are no parameters be optimized. For metric learning, the distance between the feature value and the prototype [23] is calculated to determine which category the query sample belongs to. The concept of prototype comes from the prototype network [23], and the most common definition of prototype is the average of each category’s features. To output the labels of query set, methods usually calculate the metric distance between query set and each prototype.

This paper chooses the local feature matching classifier [47]. This method has two advantages. First, for other metric learning methods, the extracted features need to be pooled that destroy the original spatial information of the features. The local feature matching classifier directly takes the extracted high-dimensional features for classification. Secondly, the local feature matching method can reduce the impact of occlusion or noise on classification to a certain extent that improves the robustness of the algorithm. The calculation process of the local feature matching classifier is as follows, as shown in (4):

(4) $$\sum_{x1=1}^{W}\sum_{y1=1}^{H}Max_{K}\left(\left\{\frac{F_{x1,y1}^{q}\cdot F_{x2,y2}^{c}}{\left|F_{x1,y1}^{q}\right|\times \left|F_{x2,y2}^{c}\right|}|1⩽x2⩽W,1⩽y2⩽H\right\}\right)$$

Among them, $F^{q}$ and $F^{c}$ respectively represent the feature of query set and the prototype, and *H* and *W* are the length and width of the feature map. $Max_{K}\left(\right)$ represents the function that is to select top *K* maximums. The classifier regards each pixel of the feature map as a local feature of the image. The calculation process of (4) is to traverse all local features on $F^{q}$. Calculate the cosine distance between the local features on $F^{q}$ and all the local features on $F^{c}$. The maximum *K* values summation is selected as the matching value, and the final summation of all matching values is the metric distance between $F^{q}$ and $F^{c}$. The larger the metric distance value represents, the closer $F^{q}$ and $F^{c}$ is. When all the category prototypes are traversed, the category with the largest metric value is selected as the category of the query image $F^{q}$.

#### 2.2.4. Parameters Optimizer

The meta-learning method chosen in this paper contains the following parameters: the CNN parameters in RESNET18, the task adaptive encoder parameters, and the FILM layer parameters. Among them, the parameters of RESNET18 are pre-trained and do not participate in the update, and the parameters of the FILM layer are generated by the task encoder. Therefore, for the LFM-CNAPS, it is the parameters in the task adaptive encoder that need to be trained and updated. The parameter update is reflected in two parts. First, during the meta-training process, the parameters in the task adaptive encoder are updated through back propagation. Secondly, during the meta-test process, the parameters in the FILM layer are updated through forward propagation. The parameters optimizer is proposed for back propagation.

For the optimizer, the most important thing is the loss function and optimization method. The loss function and optimization algorithm chosen in this paper are cross entropy loss [48] and Adam algorithms [49]. The cross entropy loss is calculated as (5):

(5) $$\sum y_{c}log\left(p_{c}\right)$$

where *y* represents the category label, *c* represents the category name, and *p* represents the predicted probability. If the query image this time is of category *c*, then the value of $y_{c}$ is 1; otherwise, it is 0. For the prediction result of the algorithm, various probabilities $p_{c}$ are obtained through the sigmoid activation function. In summary, the cross-entropy loss obtains a loss value from the label predicted by the model and the actual label.

### 2.3. Task Activation Mapping

For deep learning, most algorithms are black box. They reduce the loss through back propagation and improve the test accuracy through a large number of samples. However, deep models are not easy to visualize and could not give the basis of classification results. For CNNs, there have been many studies on visual explanations [50].

The TAM algorithm proposed in this paper is modified on the basis of the Grad-CAM [50]. The Grad-CAM process is as follows: first, a test image is needed as input, and the classification probability is obtained through the trained network. Grad-CAM will select the channel where the back propagation is located through the label. When the back propagation reaches the last layer of the CNNs, Grad-CAM would record the parameter gradient of the last layer. The gradient tensor will be averaged in the channel direction, and a one-dimensional variable whose length is the number of channels will be obtained. Grad-CAM would multiply the one-dimensional variable with the intermediate variable of the last layer to obtain the activated intermediate variable. The intermediate variables will be averaged in the direction of the feature map to obtain a two-dimensional activation layer. Grad-CAM will convert the two-dimensional activation layer mapping from 0 to 255 into a heat map. The heat map will be mapped to the input image to get a visual CNN heat map. Grad-CAM obtains the influence of various features by the degree of the convolution gradient. The brighter the red in the figure, the greater this part of the feature effect on the result.

**Figure 2:**
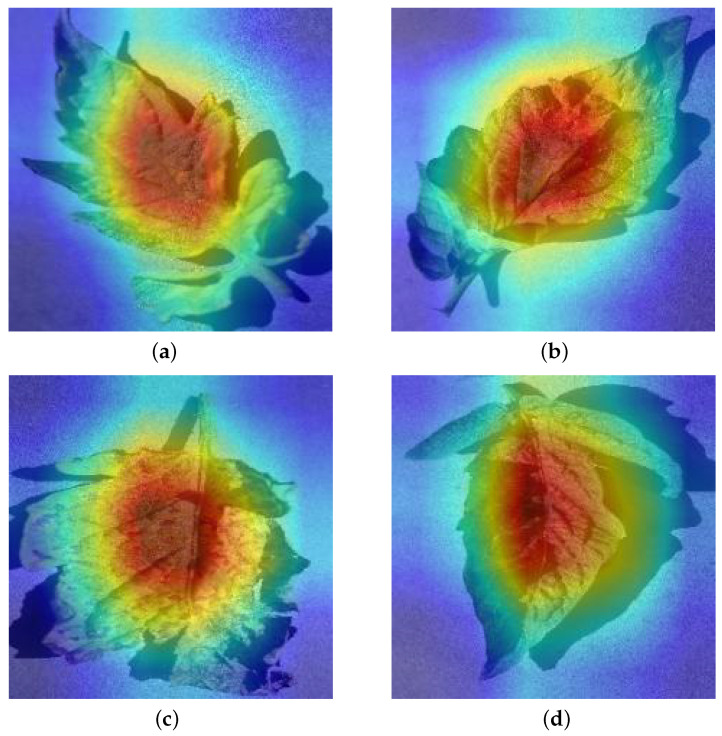
Visual explanations of tomato disease. (**a**) Leaf mold; (**b**) Late blight; (**c**) Septoria leaf spot; (**d**) two spotted spider mite.

However, for the method in this paper, the use of Grad-CAM has been restricted. Since the classifier does not contain parameters, the back propagation starts directly from the last layer of the CNNs and the parameters updated by back propagation are part of the task encoder. TAM is proposed based on the Grad-CAM. It can be known from the Grad-CAM that the pooled gradient one-dimensional variable needs to be obtained, and, from (3), γ generated by the encoder is such a variable. For the task encoder, its function is to generate parameters through task features, and interfere with the intermediate variables of RESNET18. Therefore, the γ of the last layer of CNNs is chosen to average the intermediate variables in the direction of the feature map to obtain the two-dimensional activation layer. Then, TAM will perform its mapping to get the CNN heat map. Compared with Grad-CAM, TAM does not choose categories for gradient transformation but task features. Secondly, Grad-CAM is done through back propagation gradients and TAM is through forward propagation. Visual explanations of tomato disease output by TAM are shown in Figure 2.

## 3. Results

We evaluate LFM-CNAPS on the Miniplantdisease-Dataset family of datasets, demonstrating improvements by ablation experiment. Two prediction visual explanations are also given.

### 3.1. Performance of Plant Disease Detection

We train LFM-CNAPS on the Meta-Dataset and Miniplantdisease-Dataset, evaluate it on the Miniplantdisease-Dataset and PlantVillage-Dataset. To investigate the performance of LFM-CNAPS proposed, six comparison algorithms are adopted. First, the deep learning method [2] composed of CNNs and a fully connected layer is chosen (RESNET18 + FC). To control variables, CNNs use RESNET18 that is the same as LFM-CNAPS. Next, the model composed of RESNET18 and local feature matching classifier is chosen (RESNET18 + LFM) to show the effect of task adaptive processes of LFM-CNAPS. Finally, four few-shot learning methods are adopted including: MatchingNet [29], ProtoNet [23], Simple-CNAPS [25], and Meta Fine-Tuning [27]. The information of machine specifications and time cost is shown in Table 1.

The training results of the Meta-Dataset are shown in Table 2. Table 2 is the result of the method performance on Meta-Dataset which was also trained on Meta-Dataset. Among them, cifar10 and cifar100 are not included in the training dataset and are only used for testing. The results in Table 2 can reflect the performance of the approach on general classification tasks. These include classification of animal species, classification of objects and tools, and classification of handwritten fonts. The task format of the Meta-Dataset is not fixed. Through 110,000 times of training, the algorithm has a better classification effect.

The training results of the in-domain Miniplantdisease-Dataset are shown in Table 3. A total of 20,000 tasks were randomly generated, including 60 types of plant diseases. The names of various types of plants, the number of their diseases, and the number of corresponding samples are declared in the table. The data sources are distinguished in the table. It can be seen that the two datasets do not contain the same plant categories. Each plant of Apple foliar disease has only two categories: healthy and diseased. The number of plant diseases in PlantVillage is relatively random, as many as 10 and as few as one. Through cross-domain dataset training, the model can be more robust. Secondly, from the perspective of sample size, the sample size of Apple foliar disease is much smaller than that of PlantVillage. The unbalanced sample distribution is more practical for application because there is no absolutely balanced sample in reality, and most plant classification samples are random. From the results, after 20,000 trainings, the average accuracy of the algorithm reached 97.5%.

Table 4 shows the test results of out-of-domain Miniplantdisease-Dataset. Out-of-domain and in-domain datasets do not contain the same plant diseases. Testing the algorithm through untrained plant diseases can better reflect the robustness of the algorithm. The accuracy of algorithms for newly emerged plant diseases is also more practical. The out-of-domain datasets include 12 plant disease categories and 600 random tasks. LFM-CNAPS has an average accuracy rate of 93.3% on out-of-domain dataset.

Table 5 shows ablation studies of LFM-CNAPS on an Out-of-Domain Miniplantdisease-Dataset. Our model mainly includes conditional adaptive feature extractor and local feature matching classifier components. A conditional adaptive feature extractor extracts meaningful features via forward propagation, which helps the model learn those features even in the few-shot dataset. A local feature matching classifier replaces the fully connected layers with metric learning to avoid overfitting which deeply hurts the performance of the neural network. The result of ablation experiments is shown in Table 5. LFM-CNAPS with only conditional adaptive feature extractor holds 86.1% accuracy and LFM-CNAPS with only a local feature matching classifier reaches 85.2% accuracy. However, LFM-CNAPS with the two components has 93.9% accuracy.

The test results of PlantVillage are shown in Table 6, which contains a total of 38 plant diseases. Although some plant disease categories in PlantVillage are used for meta-training, the test pictures are different from the training pictures. The number of categories and the number of samples are more than the first test. Therefore, the test on PlantVillage is more challenging than the first test, and the average accuracy of LFM-CNAPS is 89%.

### 3.2. Visual Explanations

**Figure 3:**
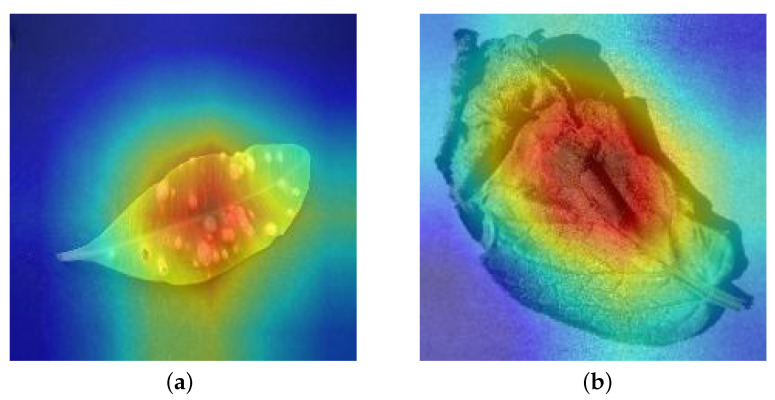
LFM-CNAPS classification basis is visualized by TAM. (**a**) the sample of diseased Alstonia Scholari; (**b**) the sample of potato with late blight.

Abnormal phenotype can be caused by either abiotic or biotic stress. The former is caused, for instance, by lack or excess of nutrients or water [51]. The latter can be caused by fungi, bacteria, and viruses. The typical symptomatology of (abiotic or biotic) stress includes discoloration, necrosis, decay, wilting, and atypical forms. Most of the existing deep learning methods for plant disease classification pay more attention to the test accuracy, and do not pay much attention to the classification basis. However, for practical application, a reasonable classification basis is more convincing and more acceptable. While our method gives the classification results, it also can save the classification’s heat map by TAM technology. As shown in Figure 3, (a) is a sample map of Alstonia Scholari affected by pests, and (b) is a sample map of potato with late blight. The red part is the part that the algorithm pays more attention to, and it is also the core part that affects the classification result. For (a), the red part mainly appears near the wormhole, and, for (b), the red part is also where the leaves turn yellow and wither. Although it is impossible to give a detailed description of the causes of plant diseases, the focus of a heat map can be used to visualize the parameters of the CNNs, and the interpretability of the black box network can be improved to a certain extent.

## 4. Discussion

Pests and diseases seriously threaten crop yields, leading to food shortages, e.g., more than 800 million people do not have adequate food; 1.3 billion live on less than $1 a day and at least 10% of global food production is lost to plant disease [19]. In order to combat the losses, the emerging plant disease needs to be detected before it has a large-scale impact on crop growth. This paper intends to propose a method that can detect plant diseases with few samples. The results showed that LFM-CNAPS proposed has an average accuracy of 93.9% on detecting unseen plant disease with only 25 annotated examples. The method RESNET18 + FC based on deep learning [10] only has an accuracy of 20.0%. More specifically, when classifying the five diseases of tomato: bacterial spot, early blight, healthy, late blight, and leaf mold, the performance of LFM-CNAPS is much better than the other two methods. LFM-CNAPS can give visual explanations through TAM, similar to optical observation of the symptoms on plant leaves. As show in Figure 3, Alstonia Scholari’s wormhole is presented. However, due to complexity, even experienced agronomists often fail to successfully diagnose specific diseases [2]. For example, it is difficult for people to distinguish the corn with northern leaf blight from the corn with gray leaf spot. LFM-CNAPS adaptively adjusts and extracts the potential differences between the two categories by task adaptive processes. This feature will be more abstract rather than simple geometric features. On a commercial scale, evidently, a capital investment is initially required for adopting the employed approach [52]. Nevertheless, the wide-ranging large-scale commercial applications can provide high returns through considerable improvements in process enhancement and cost reduction.

Limitations of the study are due to a single task format. All tests are based on using 25 samples for plant disease detection. Recommendations for further research are to deal with a different scale of samples for better classification results.

## 5. Conclusions

This paper proposed LFM-CNAPS to solve few-shot plant disease detection and made the following contributions: first, a Miniplantdisease-Dataset suitable for meta-learning is provided, including two public datasets, 60 plant disease categories. Secondly, the LFM-CNAPS proposed is evaluated on the Miniplantdisease-Dataset, with an accuracy rate of 93.9% . Finally, TAM was proposed for CNN visualization. Without affecting the classification results and time cost, the classification heat map is saved to realize visual explanations.

## Figures and Tables

**Table 1:** Machine specifications and time cost.

Name	Value
Video Memory	11G
Graphics	NVIDIA GeForce GTX 1080 Ti
Processor	Intel(R) Xeon(R) CPU E5-2640
Operating system	Windows 10 Home 64
Training time	17,548.73 s
Test time	57.36 s

**Table 2:** LFM-CNAPS test accuracy on the Meta-Dataset.

Dataset Name	Accuracy (%)
ilsvrc 2012	55.0+/−1.0
omniglot	92.0+/−0.6
aircraft	82.4+/−0.6
cu birds	74.3+/−0.8
dtd	65.3+/−0.7
quickdraw	75.5+/−0.8
fungi	48.0+/−1.1
Vgg flower	89.4+/−0.5
Traffic sign	68.2+/−0.7
mscoco	51.1+/−1.0
mnist	93.3+/−0.4
cifar10	71.1+/−0.7
cifar100	57.3+/−1.0

**Table 3:** LFM-CNAPS train results on in-domain Miniplantdisease-Dataset.

Species	Number of Plant Diseases	Number of Samples
Apple foliar disease		
Alstonia Scholaris	2	433
Arjun	2	452
Bael	2	266
Chinar	2	223
Gauva	2	419
Jamun	2	624
Jatropha	2	257
Lemon	2	236
PlantVillage		
Apple	4	7169
Blueberry	1	1816
Cherry	2	3509
Corn	4	7316
Grape	4	7222
Orange	1	2010
Peach	2	3566
Pepper	2	3901
Potato	2	3763
Tomato	10	18,345
Number of training steps		Training accuracy (%)
10,000		97.0
20,000		97.5

**Table 4:** LFM-CNAPS test results on an Out-of-Domain Miniplantdisease-Dataset.

**Plant State**	**Number of Samples**
Mango diseased	265
Mango healthy	170
Pomegranate diseased	272
Pomegranate healthy	287
Pongamia Pinnata diseased	276
Pongamia Pinnata healthy	322
Potato Late blight	1939
Raspberry healthy	1781
Soybean healthy	2022
Squash Powdery mildew	1736
Strawberry healthy	1824
Strawberry Leaf scorch	1774
**Method**	**Accuracy (%)**
RESNET18 + FC	20.0+/−0.5
MatchingNet	19.5+/−0.5
ProtoNet	20.5+/−0.6
RESNET18 + LFM	85.2+/−0.7
Simple-CNAPS	92.5+/−0.4
Meta Fine-Tuning	91.14+/−0.5
LFM-CNAPS	93.9+/−0.4

**Table 5:** Ablation studies of LFM-CNAPS on an Out-of-Domain Miniplantdisease-Dataset.

Feature Extractor	Classifier	Accuracy (%)
		20.0+/−0.5
✓		86.1+/−0.6
	✓	85.2+/−0.7
✓	✓	93.9+/−0.4

**Table 6:** LFM-CNAPS test results on a PlantVillage-Dataset.

**Plant Category**	**Number of Plant Diseases**	**Number of Samples**
Apple	4	1943
Blueberry	1	454
Cherry	2	877
Corn	4	1829
Grape	4	1805
Orange	1	503
Peach	2	891
Pepper	2	975
Potato	3	1426
Raspberry	1	445
Soybean	1	505
Squash	1	434
Strawberry	2	900
Tomato	10	4585
**Method**		**Test accuracy (%)**
RESNET18 + FC		19.8+/−0.5
RESNET18 + LFM		81.7+/−0.7
LFM-CNAPS		89.0+/−0.5

## Data Availability

The dataset can be obtained from https://www.kaggle.com/vipoooool/new-plant-diseases-dataset (accessed on 14 July 2021) and https://www.kaggle.com/c/plant-pathology-2020-fgvc7 (accessed on 14 July 2021) .
